# Case Series Analysis of No-Touch Graft Configurations in Coronary Artery Bypass Grafting: Comparative Outcomes

**DOI:** 10.3390/jcm15208125

**Published:** 2026-10-20

**Authors:** Dragana Radoičić, Jelena Kljajević, Ivan Nešić, Matija Furtula, Dalibor Dragišić, Aleksandar Pavlović, Milica Ivanović, Slobodan Tomić, Marko Kaitović, Natalija Arnautović, Armin Šljivo, Aleksandar Milutinović

**Affiliations:** 1 Institute for Cardiovascular Disease “Dedinje”, 11000 Belgrade, Serbia; jelenakljajevic@gmail.com (J.K.); ivan.nesic@gmail.com (I.N.); furtulam1205@gmail.com (M.F.); milicaivanovicks@gmail.com (M.I.); bobantomic99@gmail.com (S.T.); marko.kaitovic@gmail.com (M.K.); gnatalija@ymail.com (N.A.); 2 Hospital Dragisa Misovic, 11040 Belgrade, Serbia; dalibor.dragisic@gmail.com; 3 Department for Cardiology, University Hospital Medical Center Bežanijska kosa, 11080 Belgrade, Serbia; dracapavlovic@gmail.com; 4 Department for Cardiovascular Surgery, Clinical Center of University of Sarajevo, 71000 Sarajevo, Bosnia and Herzegovina; 5 Department for Cardiovascular Surgery, University Children’s Hospital, “Tiršova”, 11000 Belgrade, Serbia; aleksandarm1982@yahoo.com

**Keywords:** coronary artery disease, CABG, cardiac surgery, no-touch vein harvesting, Serbia

## Abstract

**Introduction**. This study aims to evaluate the outcomes of three distinct configurations of No-Touch (NT) grafts used in coronary artery bypass grafting, focusing on the safety and efficacy of these techniques. **Methods**. Nine cases were analysed and categorized into three groups: Coronaro-Coronary NT Left Internal Thoracic Artery (LITA) non-aortic composite grafts; NT-LITA ‘Y’ composite grafts, and a Posterior Descending-Obtuse Marginal-Aorta NT vein graft. **Results**. In Group 1, no traditional proximal anastomosis was performed. The NT vein graft was distally anastomosed to the obtuse marginal branch in an end-to-side manner, followed by side-to-side anastomosis to the Ramus Intermedius (RI) or Diagonal, and a final end-to-side connection to the Ramus Interventricularis Anterior (RIA). In Group 2, where NT-LITA ‘Y’ composite grafts were utilized, the first graft was placed end-to-side and the second side-to-side, addressing either two marginal branches or the OM and RI branches. Proximal anastomoses were occasionally performed to the LITA or aorta. In Group 3, a sequential anastomosis was conducted, with the NT vein graft connected from the right posterior descending artery in an end-to-side manner, followed by a side-to-side connection to the OM branch. Patients had patent No-Touch vein grafts on their follow-up MSCT, without any post-surgery complications, except 2 cases where one had occluded marginal branches, and one LITA-RIA anastomosis. **Conclusions**. Different No-Touch graft configurations demonstrate high patency rates and a favorable safety profile. Overall, graft patency was preserved in most patients during follow-up, although several anastomotic occlusions were observed. These findings suggest NT grafts are a viable alternative to traditional methods, warranting further research into long-term outcomes and surgical refinements.

## 1. Introduction

The advantages of No-Touch (NT) vein grafts over conventional vein (CV) grafts in modern coronary surgery have been well-documented and validated through both short- and long-term studies [1], leading to their classification as a IIb recommendation in the guidelines for coronary revascularization [2]. The superior patency of NT vein grafts underscores the technique’s histological and molecular benefits, which better preserve tissue integrity compared to conventional methods [3]. Conventional vein grafts show structural deterioration and higher graft occlusion rates even in short-term follow-up [4], with long-term studies [5], such as those by Souza et al., revealing increasingly pronounced differences after 20, 25, or 30 years. The superiority of NT vein grafts in coronary surgery is largely due to the pedicle’s richness in protective molecules like nitric oxide and leptin, which enhance vasodilation [6,7]. The intact adventitia preserves the vasa vasorum, crucial for nourishing the vein wall—an advantage not seen in CV grafts [8,9]. Additionally, the pedicle prevents vein spasm, while the NT technique shields the vein from endothelial injury caused by syringes and barotrauma. Despite its benefits, the technical advantages of NT vein grafts, particularly their versatility in surgical applications, remain underexplored. NT grafts provide greater options for creating sequential anastomoses and act as external stents, absorbing vein wall stress from arterial circulation. The absence of kinking, a documented benefit, also warrants further discussion as we seek more innovative and durable solutions in coronary surgery [5,10]. By employing sequential and composite anastomoses, we can reduce leg trauma, minimize manipulation of the aorta, and achieve comparable results with shorter vein grafts and reduced leg incision length. This study aims to evaluate the outcomes of three distinct configurations of NT grafts used in coronary artery bypass grafting (CABG), focusing on the safety and efficacy of these techniques.

## 2. Materials and Methods

The research was conducted at the Department of Cardiovascular Surgery, Institute for Cardiovascular Disease “Dedinje,” Belgrade, Serbia, and aims to contribute to the understanding of innovative grafting techniques in CABG. This case series study evaluates nine cases classified into three distinct categories: Group 1 (“Drina Bridge Technique”) encompasses cases utilizing Coronaro-Coronary No-Touch (NT) Left Internal Thoracic Artery (LITA) non-aortic composite grafts; Group 2 comprises NT-LITA ‘Y’ composite grafts; and Group 3 includes a single case employing a Posterior Descending-Obtuse Marginal-Aorta NT vein graft.

Indications for CABG were based on invasive coronary angiography, defining significant coronary stenosis as a 70% reduction in lumen diameter or a functional flow reserve (FFR) value of less than 0.75 for borderline stenosis. All patients underwent echocardiography as part of the comprehensive preoperative evaluation, assessing heart function (ejection fraction and myocardial contractility), valve function, and the morphology of the heart and great vessels.

Some cases were pre-planned, while others were performed as bailout strategies due to insufficient graft length, calcified aorta, or small coronary targets. Shorter skin incisions were used to reduce leg trauma, accommodating comorbidities or the surgeon’s preference, while ensuring complete revascularization through sequential anastomoses and shorter vein grafts. All patients were elective and asymptomatic, except for one with acute coronary syndrome (ACS), who did not survive.

### 2.1. Inclusion and Exclusion Criteria

This study included patients undergoing isolated CABG based on the following criteria: (1) vein graft harvesting performed by a cardiac surgeon with over 100 vein grafts annually, (2) CABG procedures involving at least one NT great saphenous vein graft, and (3) preoperative ultrasound vein mapping showing an acceptable vein diameter of 2–5 mm.

Exclusion criteria were: (1) combined cardiac surgical procedures, (2) redo CABG, (3) failed percutaneous coronary intervention (PCI) with hemodynamic instability, (4) severe peripheral vascular disease, (5) scheduled total arterial revascularization, (6) multiple arterial grafts for coronary revascularization, (7) history of vein stripping, and (8) dilated or varicose great saphenous veins identified by ultrasound vein mapping.

### 2.2. Preoperative Ultrasound Great Saphenous Vein Mapping

Vein mapping is performed one day prior to the CABG procedure, except for patients with ACS, who undergo mapping immediately before surgery. The vein diameter is assessed at multiple sites, with an acceptable range of 2 to 5 mm for inclusion in the study. Superficial veins (approximately 4 mm or closer to the skin) are deemed unsuitable for the NT technique due to the complexities of pedicle creation and the need to minimize tissue trauma. Large tributaries, varicosities, and duplicated vein systems are also identified and marked with skin landmarks to aid in surgical planning.

### 2.3. Harvesting Technique

A longitudinal skin incision was made with a scalpel, and subcutaneous tissue was dissected using low-power diathermy to preserve fascia and perivascular tissues. The vein was harvested with a 1 cm-wide fat pedicle, and side branches were ligated with 4-0 silk sutures or metal clips. After achieving the required length, the vein was removed, and a cannula was inserted into the distal end. It was briefly flushed with heparinized saline while keeping the opposite end open and then stored on ice. The vein cannula was connected to a side line from the arterial cannula in the ascending aorta, with heparinized blood used for distension and identifying bleeders, maintaining pressure at 80–100 mmHg and clipping any leaking side branches. Subcutaneous incisions were closed with continuous Vicryl sutures (Vicryl suture—Ethicon Inc., Somerville, New Jersey, USA), and skin incisions were closed with intradermal or interrupted sutures per the surgeon’s preference.

### 2.4. CABG Procedure and Anastomosis Technique

Patients underwent CABG under general anaesthesia, employing cardiopulmonary bypass and systemic heparinization, with a single clamp technique and myocardial protection achieved through antegrade crystalloid or blood cardioplegic solution. The types of proximal anastomoses varied depending on the sequential and/or composite graft configurations.

In Drina Bridge Technique Group (Figure S1), no traditional proximal anastomosis was performed. The NT vein graft was sewn distally to the marginal branch (OM) in an end-to-side manner, then to the Ramus Intermedius (RI) or Diagonal (DG) in a side-to-side fashion, and finally to the Ramus Interventricularis Anterior (RIA) in an end-to-side configuration. LITA was anastomosed end-to-side to the NT vein strictly at the hood of the NT-RIA anastomosis to ensure proper alignment and to avoid potential complications related to vein valves.

In the NT-LITA ‘Y’ vein graft group (Figure S2), proximal anastomoses were occasionally made to the LITA or aorta when bypassing two marginal branches (with the first graft end-to-side and the second side-to-side) or addressing the OM and RI branches. In some cases, a short segment of the NT vein was sewn onto the Diagonal (DG) artery, followed by a “Y” composite anastomosis with the proximal LITA.

In the sequential anastomosis case (Figure S3), a proximal anastomosis was performed to the aorta after the NT vein graft was connected end-to-side from the right posterior descending artery (PD) to the OM branch in a side-to-side fashion.

Distal anastomoses of the grafts were performed using standard techniques, either end-to-side or side-to-side. The anastomosis between the LITA and the RIA was consistently executed with 8-0 Prolene sutures. Anastomoses involving marginal, diagonal, or intermediate branches used either 7-0 or 8-0 Prolene sutures, depending on case requirements. Proximal anastomoses of vein grafts to the aorta were secured with 6-0 Prolene sutures, while those to the LITA used 8-0 Prolene sutures. In the Drina Bridge Technique, the LITA was directly anastomosed to the vein at the hood of the RIA-NT end-to-side anastomosis with 8-0 Prolene sutures.

### 2.5. Follow-Up

All patients were contacted for follow-up six months to one year after the procedure, depending on hospital and healthcare system capabilities. Follow-up imaging was conducted using either multislice computed tomography (MSCT) coronary angiography or conventional coronary angiography, with MSCT providing greater patient comfort as it does not require hospitalization. A clinical examination was performed, and major adverse cardiovascular and cerebrovascular events (MACCE), including angina pectoris, periprocedural myocardial infarction, repeat revascularization, transient ischemic attacks, and strokes, were recorded.

Follow-up imaging was performed using a Philips Brilliance CT scanner, with MSCT coronary angiography utilizing retrospective ECG-gated contrast-enhanced cardiac CT at a slice thickness of 0.67 mm. Data visualization techniques, including volume rendering for 3D representation and multi-planar reconstruction for oblique plane visualization, were employed. Graft stenosis and occlusion were assessed by two highly experienced radiologists.

## 3. Results

This case series, conducted at the Department of Cardiovascular Surgery, Institute for Cardiovascular Disease “Dedinje,” Belgrade, Serbia, evaluates nine cases categorized into three distinct groups. All data regarding patient Characteristics, surgical techniques, and MSCT Follow-up outcomes in CABG with NT Grafts are presented in Table 1.

### 3.1. Coronaro-Coronary NT-LITA Non Aortic Composite Graft—Drina Bridge Technique

This group comprised four patients, three of whom underwent elective procedures. All elective cases demonstrated patent anastomoses on follow-up, with patients remaining asymptomatic and achieving full recovery postoperatively. Postoperative coronary angiographic findings of the No-Touch (NT) vein graft are presented in Figure S4.

The fourth patient, who presented with ACS, had graftable vessels with challenging dimensions: the right posterior descending (PD) artery, OM branch, and ramus intermedius (RI) had diameters around 1.1–1.2 mm, as verified with a probe. Only the ramus interventricularis anterior (RIA) had an acceptable diameter of approximately 1.5 mm. Unfortunately, the patient did not survive. A recoronarography was performed due to clinical deterioration and elevated troponin levels, revealing occlusion of the aortic-to-posterior descending (Ao-PD) graft and the OM and RI anastomoses from the Coronaro-Coronary composite graft, with only the RIA remaining patent. Despite ICU care and readmission, the patient passed away.

### 3.2. NT-LITA ‘Y’ Vein Graft

This group consisted of four patients, all of whom were asymptomatic, fully recovered, and demonstrated preserved functionality postoperatively. In two patients with sequential anastomoses connected proximally to the aorta, all graft anastomoses were confirmed patent upon follow-up assessment. Postoperative coronary angiographic findings of the No-Touch (NT) vein graft are presented in Figure S4.

One patient with a “Y” composite graft had two marginal branches supplied by the LITA, which resulted in a patent LITA-RIA anastomosis; however, both marginal branches were occluded. Another patient had a proximal anastomosis of a NT vein graft—previously used for two sequential anastomoses—connected to the LITA, resulting in patent anastomoses, although the blood supply was derived from the native coronary circulation.

### 3.3. Sequential Anastomosis NT Vein Graft; PD-OM-Aorta NT Vein Graft

The patient remained asymptomatic, with both anastomoses initially appearing patent. However, occlusion of the LITA-RIA anastomosis occurred despite the absence of technical complications during the surgical procedure and no reported postoperative issues, apart from the notably small diameters of the LITA and RIA. This finding was further supported by the patient’s medical history both during hospitalization and following discharge. Postoperative coronary angiographic findings of the No-Touch (NT) vein graft are presented in Figure S4.

## 4. Discussion

To the best of the authors’ knowledge, this study is among the first to investigate the utilization of various NT techniques in CABG. We present a series of nine cases categorized into three distinct groups: Drina Bridge Technique- Coronaro-Coronary NT LITA non-aortic composite grafts, NT-LITA ‘Y’ composite grafts, and a single case involving a Posterior Descending-Obtuse Marginal-Aorta NT vein graft. Follow-up assessments revealed that all Drina Bridge Technique-Coronaro-Coronary NT-LITA grafts maintained patency. Within the NT-LITA ‘Y’ group all grafts maintained patency except in one patient exhibited a patent LITA-RIA anastomosis; however, both marginal branches were occluded and patient was angina free. Lastly, in the sequential anastomosis group, the LITA-RIA anastomosis was found to be occluded even though construction PD-Om was pateent and patient was angina free.

The application of Drina Bridge Technique (Coronaro-Coronary Composite NT-LITA grafts), though sometimes considered unconventional or high-risk, is both effective and safe when used in carefully selected clinical contexts. This approach is particularly advantageous in situations where the aorta is calcified and unsuitable for proximal anastomosis, or when unexpected calcification is discovered intraoperatively. It is also beneficial in cases with limited graft availability, such as in redo coronary artery bypass procedures or in patients with contraindications for leg vein harvesting. Additionally, this method offers improved runoff in patients with small coronary arteries and minimizes the risk of kinking, as NT veins are less prone to this complication and are well-suited for sequential anastomoses. CV are prone to kinking, making sequential anastomoses challenging, while NT veins, due to their lack of valves, simplify the process [11]. Although radial arteries are useful, their length and smaller diameter limit their use for multiple anastomoses. NT veins facilitate easier creation of sequential anastomoses. To prevent compromising the RIA, it is essential to ensure no valve is present between the NT-RIA anastomosis and the LITA attachment site, ideally achieved by directly sewing the LITA onto the hood of the NT-RIA anastomosis for optimal valve identification and management. The NT vein graft technique is well-suited for minimally invasive CABG, where it avoids the need for a sternotomy and addresses the technical challenges of proximal anastomosis in the absence of a standard aortic anastomosis, offering a viable and advantageous solution.

Avoiding aortic manipulation during proximal anastomosis may reduce cerebrovascular event risk [12], while the beneficial properties of the mammary artery [13] help protect vein grafts within arterial circulation. The stress on grafts is lower when nourished by the mammary artery than with direct aortic anastomosis. This composite graft technique is also effective in off-pump surgeries, offering a cost-efficient alternative to the heart string device [14]. The time required for creating Drina Bridge Technique (Coronaro-Coronary NT-LITA composite grafts) is comparable to conventional surgery. For example, three bypasses using this technique involve only four anastomoses instead of the standard five. While initial graft length measurements may be time-consuming, this method could ultimately be more efficient with experience.

The integration of Y grafts using venous and mammary conduits is well-established in coronary revascularization [15], with NT veins offering significant advantages for sequential anastomoses. When the LITA provides adequate flow to sustain the coronary circulation, NT veins in Y graft configurations are particularly beneficial in situations such as: requiring a graft extension to optimize left coronary perfusion; implementing a Y graft as a strategic component of the revascularization plan; addressing a porcelain aortic root that precludes safe cross-clamping; avoiding proximal anastomosis during off-pump surgery; and facilitating minimally invasive coronary procedures [16,17].

In our series, two patients with Y grafts incorporating two sequential anastomoses on the left side (targeting either two marginal branches or a marginal and an intermediate branch) maintained complete graft patency. In contrast, two patients with proximal anastomoses to the LITA experienced occlusion or functional occlusion, likely due to minor native stenoses and significant native coronary flow. These occlusions could be attributed to technical factors, such as vein graft twisting, yet both patients remained asymptomatic. In cases where marginal branch occlusion occurred but the LITA-RIA anastomosis remained patent, patients continued symptom-free, underscoring the need for further data to assess the potential benefits of highly complex anastomoses for achieving complete revascularization, particularly regarding the LITA-RIA connection.

One of the limitations of this study is the small sample size and heterogeneity of the cases, which are inherent to the nature of a technical case series. As these findings are preliminary and hypothesis-generating rather than definitive, larger, well-designed studies with longer follow-up and larger patient populations are needed to confirm their safety, efficacy, and long-term outcomes. The absence of a control group, these findings should be considered preliminary and no definitive comparative conclusions regarding safety, efficacy, or superiority can be drawn.

Based on these findings, we recommend prioritizing Drina Bridge Technique (coronary-coronary sequential anastomoses and direct LITA anastomosis) over Y grafts with NT veins and the LITA. This strategy can notably reduce leg incision length by 15–20 cm and decrease the risk of wound healing complications, as observed in prior patient groups. For vein harvesting, we prefer the upper leg if the lower leg has minimal subcutaneous tissue. Typically, around 40 cm of vein is required for two grafts [18]. Employing NT vein grafts in Y graft configurations can further reduce incision length to 20–25 cm in specific cases. Although not universally applicable, the feasibility of this technique increases with surgical familiarity, enabling more routine implementation as proficiency grows.

## 5. Conclusions

In conclusion, this study demonstrates the efficacy of Drina Bridge Technique (Coronaro-Coronary Composite NT-LITA grafts) and NT-LITA ‘Y’ grafts in CABG, particularly for cases with calcified aortas and limited graft availability. These techniques offer high patency rates, are suitable for minimally invasive and off-pump procedures, and reduce graft kinking while facilitating sequential anastomoses. Additionally, they minimize leg incision lengths and related wound healing complications. Although not universally applicable, their feasibility improves with surgical proficiency. Further research is needed to assess long-term outcomes and optimal patient selection for these advanced grafting methods in achieving complete revascularization.

## Figures and Tables

**Table 1:** Patient Characteristics, Surgical Techniques, and MSCT Follow-up Outcomes in CABG with NT Grafts.

Group	Coronaro-coronary NT-LITA (Drina Bridge Technique)				NT-LITA Y Vein Graft				Sequential Anastomosis
Patient	1	2	3	4	5	6	7	8	9
Age	68	71	59	72	65	60	69	66	63
Sex	Male	Female	Male	Female	Male	Male	Female	Female	Male
Risk factors	DM, HL	Smoking, HT	HT, smoking	HT, DL	Obesity, HT	DM, smoking	Smoking, HT	HT, DL	Smoking, HT
Chief complaint	Chest pain	Dyspnea	Angina	Chest pain	Angina	Angina	Dypsnea	Angina	Chest pain
Operation type	Coronaro-coronary NT-LITA CABG				*NT-LITA Y vein graft CABG*				Sequential anastomosis CABG
Proximal anastomosis	8-0 Prolene	8-0 Prolene	8-0 Prolene	8-0 Prolene	6-0 Prolene	6-0 Prolene	6-0 Prolene	6-0 Prolene	6-0 Prolene
Distal anatomosis	7-0 Prolene	8-0 Prolene	7-0 Prolene	8-0 Prolene	7-0 Prolene	8-0 Prolene	7-0 Prolene	8-0 Prolene	7-0 Prolene
MSCT follow up	Optimal patency with unobstructed flow	Optimal patency with unobstructed flow	Optimal patency with unobstructed flow	N/A	Optimal patency with unobstructed flow	Patent LITA-RIA anastomosis, but both marginal branches were occluded	Optimal patency with unobstructed flow	Optimal patency with unobstructed flow	LITA-RIA anastomosis occluded

N/A—non applicable.

## Data Availability

The raw data supporting the conclusions of this article will be made available by the authors on request.

## Supplementary Materials

The following supporting information can be downloaded at: https://www.mdpi.com/article/10.3390/jcm15208125/s1. Figure S1: Drina Bridge Technique (Coronaro-coronary NT vein graft); Figure S2: NT-LITA Y vein graft; Figure S3: Sequential Anastomosis NT vein graft; Figure S4: Presentation of the NT vein graft post operatively.
