# Supplementary material for: Case Series Analysis of No-Touch Graft Configurations in Coronary Artery Bypass Grafting: Comparative Outcomes
Source: J Clin Med. 2026 Oct 20;15(20):8125. doi: 10.3390/jcm15208125 (PMC13705683; doi:10.3390/jcm15208125)
Supplement: Supplementary file 1 [file jcm-15-08125-s001.zip › jcm-4538086-supplementary.pdf]

Figure S1. **Drina Bridge Technique** (Coronaro-coronary NT vein graft)

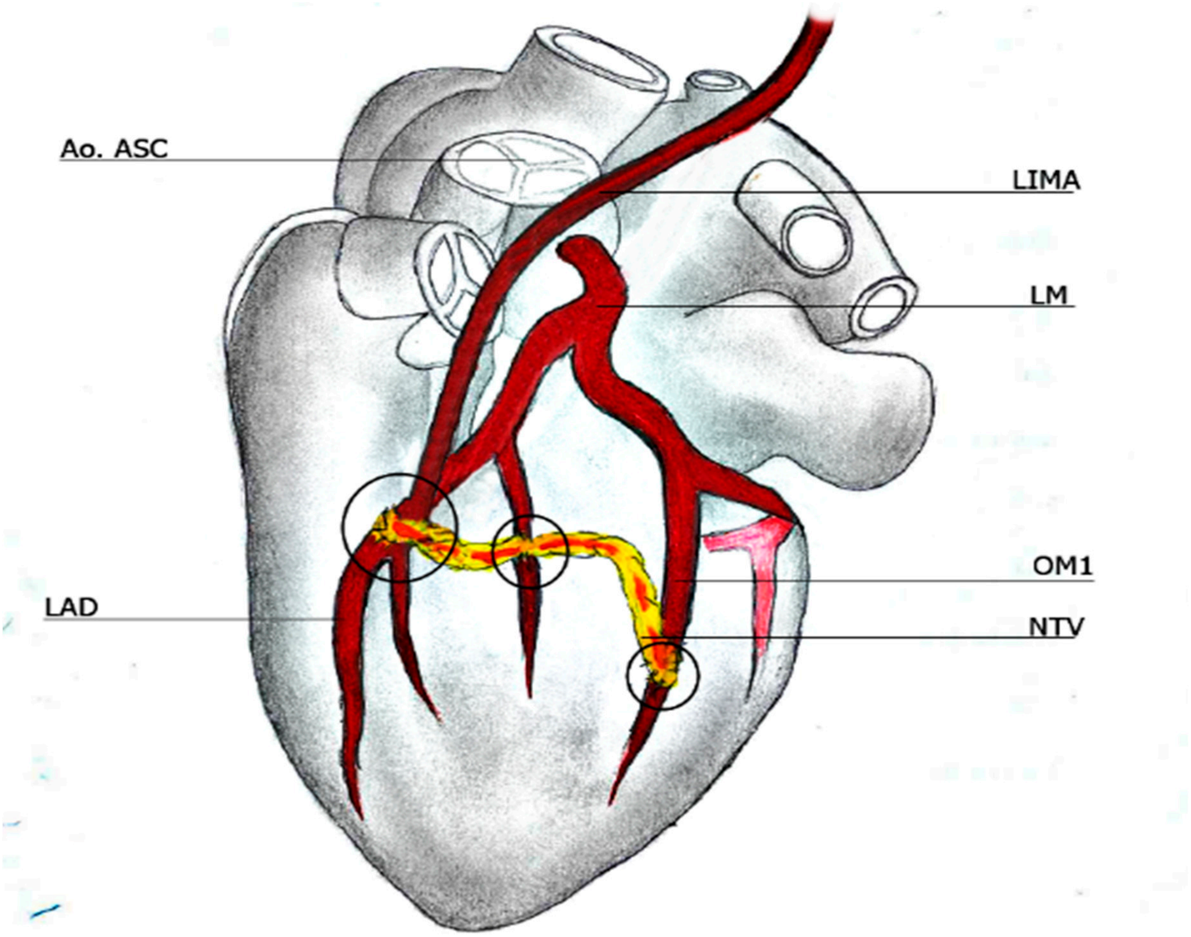

Figure S2. NT-LITA Y vein graft

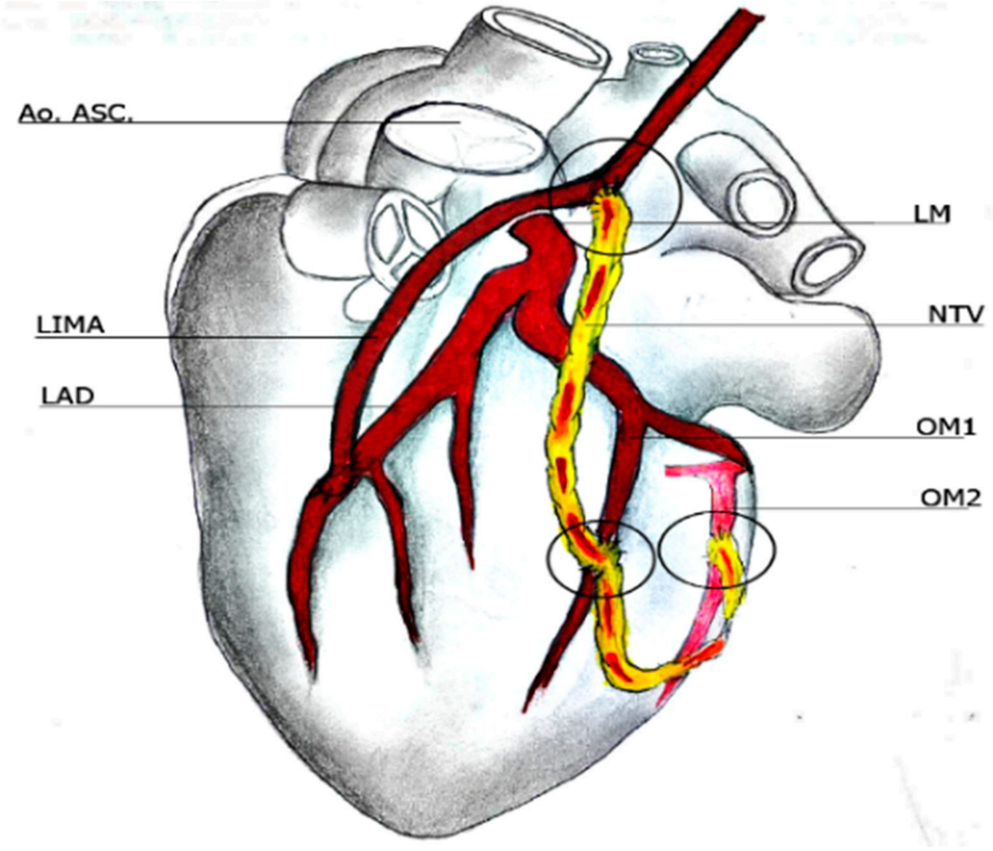

Figure S3. Sequential Anastomosis NT vein graft

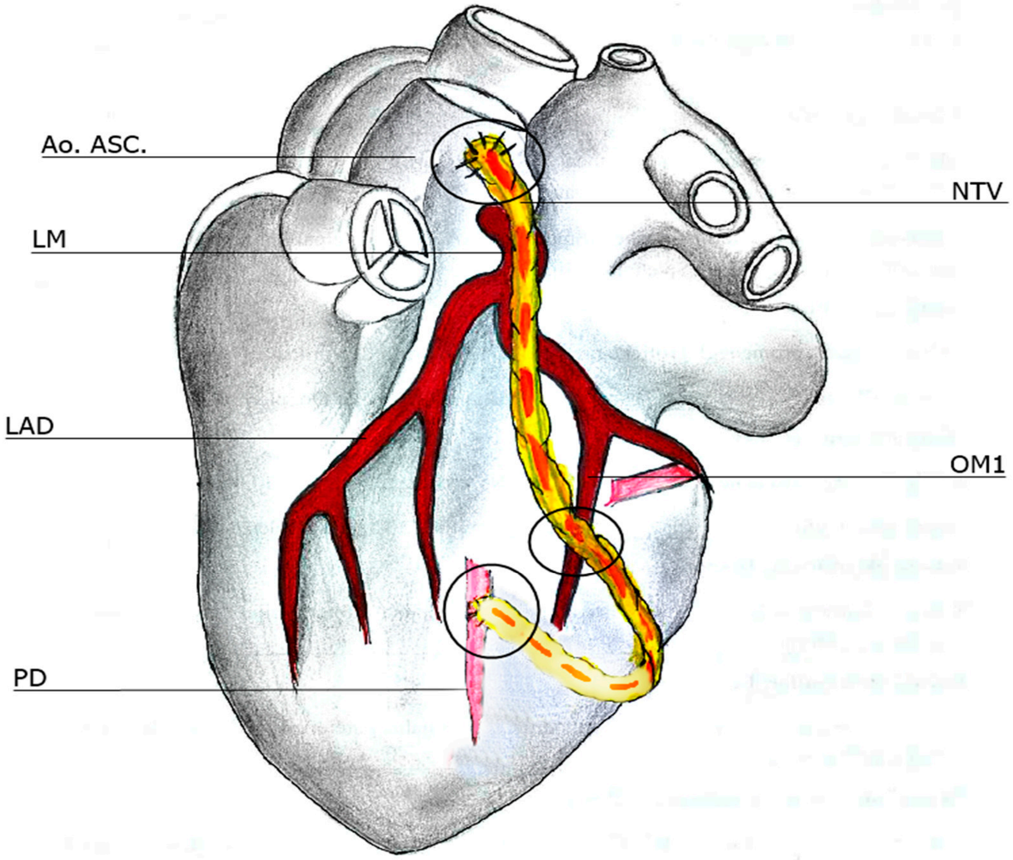

Figure S4. Presentation of the NT vein graft post operatively

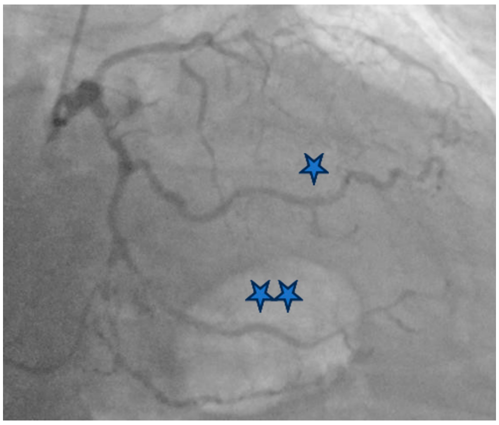

RAO/Caudal projection before revascularization.
First obtuse marginal branch
Second obtuse marginal branch

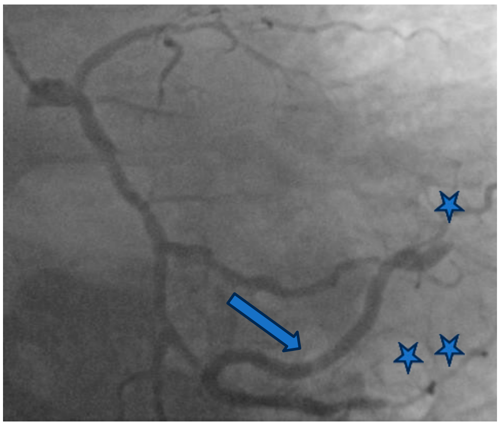

PA/Caudal projection after revascularization.
First obtuse marginal branch
Second obtuse marginal branch
No touch vain

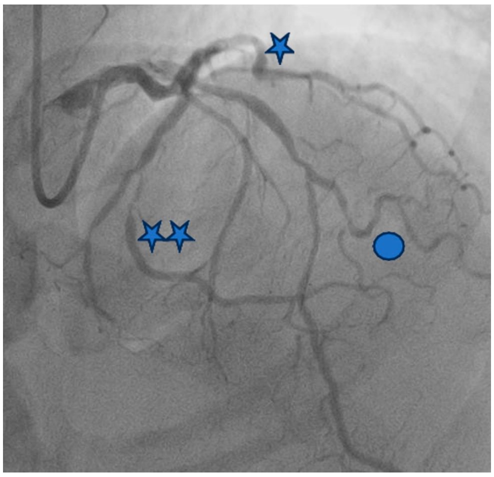

RAO/Cranial projection before revascularization.
First obtuse marginal branch
Second obtuse marginal branch
Diagonal branch

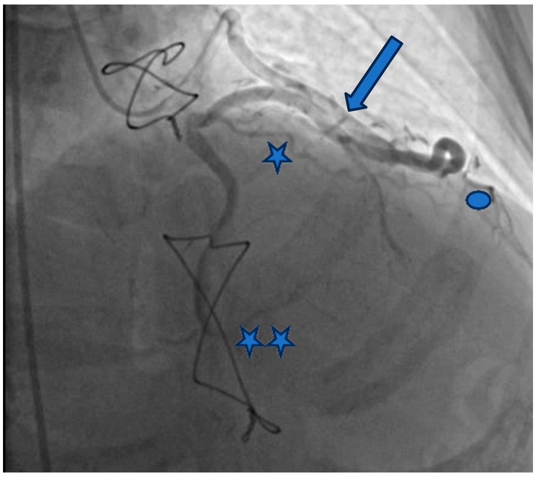

PA/Cranial projection after revascularization.
First obtuse marginal branch
Second obtuse marginal branch
Diagonal branch
No touch vain

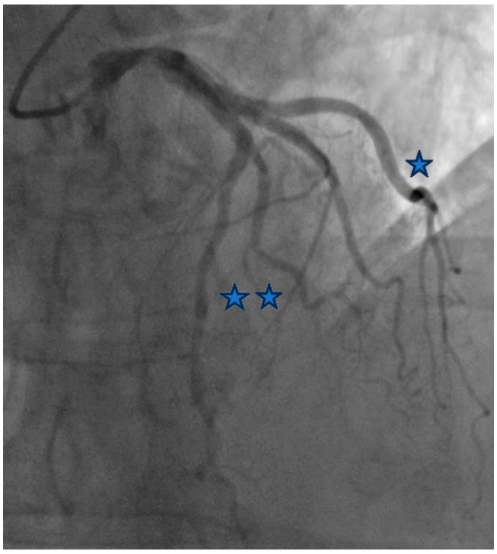

PA/Cranial projection before revascularization.
★ First obtuse marginal branch
★★ Diagonal branch

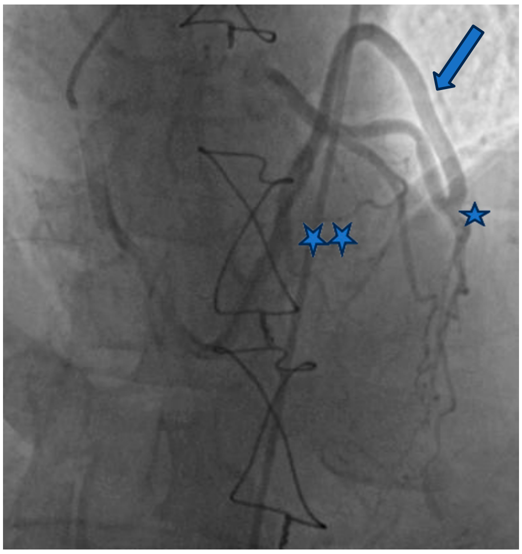

LAO/Cranial projection after revascularization.
★ First obtuse marginal branch
★★ Diagonal branch
↙ No touch vain with proximal aortic anastomosis

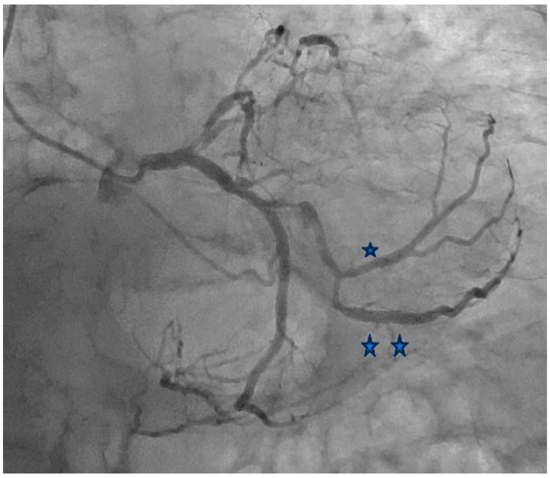

PA/Caudal projection before revascularization.
★ First obtuse marginal branch
★★ Second obtuse marginal branch

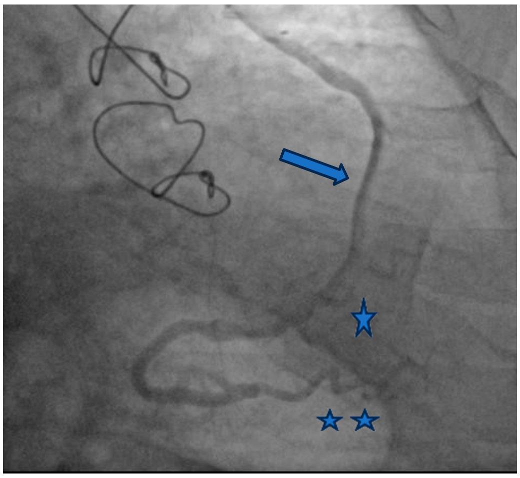

RAO/Caudal projection after revascularization.
★ First obtuse marginal branch
★★ Second obtuse marginal branch
↙ No touch vain with proximal anastomosis on aorta

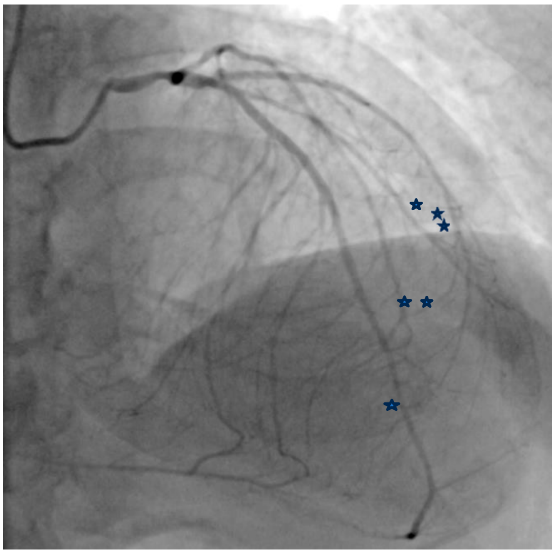

PA/Cranial projection before revascularization.

★ LAD

★★ Ramus intermedius

★★★ Obtuse marginal branch

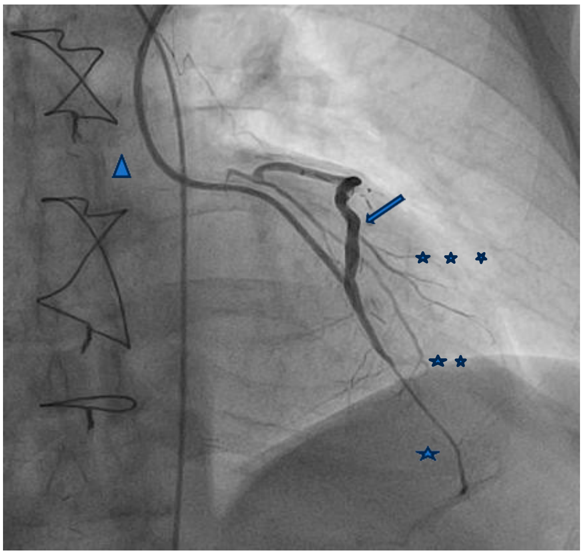

PA/Cranial projection after revascularization.

★ LAD

★★ Ramus intermedius

★★★ Obtuse marginal branch

⬇ No touch vain

▲ LITA

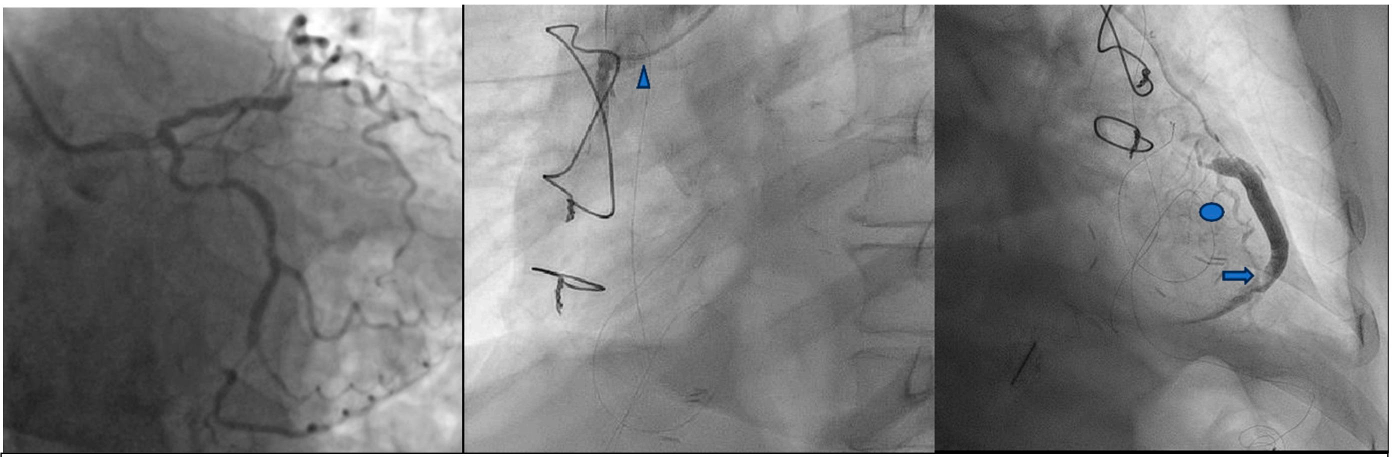

Upon examination from right to left, there is observed a diffusely diseased native circumplex artery. In this instance, a coronary-coronary artery bypass graft (OM-Rim-LAD) was conducted utilizing a no-touch vein, to which a LITA graft was anastomosed. Additionally, an aortocoronary bypass was performed employing a vein graft on the right coronary artery. However, 72 hours post-operation, acute coronary syndrome ensued, revealing thrombosis of the vein graft on the right coronary artery and thrombosis of the no-touch vein, with a prominent thrombus evident upon visualization.
